# The Struggle Against Air Pollution in African Megacities and the Hidden Problems for the Estimation of the Burden of Disease

**DOI:** 10.1002/gch2.202500108

**Published:** 2025-08-26

**Authors:** Vasileios N Matthaios, Daniel Pope, Petros Koutrakis, Christopher O Olopade, Crystal M North

**Affiliations:** ^1^ Department of Public Health Policy and Systems University of Liverpool Liverpool L69 3GB UK; ^2^ Department of Environmental Health Harvard T.H. Chan School of Public Health Boston MA USA; ^3^ Pulmonary Critical Care and Sleep Medicine. Biological Science Division and Pritzker School of Medicine University of Chicago Chicago USA; ^4^ Harvard Medical School Boston MA USA; ^5^ Pulmonary and Critical Medicine Department of Medicine Massachusetts General Hospital Boston MA USA

**Keywords:** Africa, air quality, health, megacities, sustainable development, traffic, urbanization

## Abstract

Air pollution poses a significant threat to global public health, with African megacities facing its severe consequences due to rapid urbanization, industrialization, and transportation challenges. In Africa, air pollution is responsible for 1.1 million deaths annually, with household air pollution accounting for two‐third and ambient air pollution one‐third of this burden. However, these percentages are likely to change in the near future due to the projected rapid urbanization and industrialization in the region. In the next 25 to 50 years African megacities are projected to grow rapidly and therefore experience a significant increase in air pollution‐related health risks. Poor policy prioritization, limited monitoring infrastructure and conflicting interests and priorities further complicate the problem. In this paper, the key drivers of air pollution are discussed in African megacities, including urbanization, industrialization, transportation, and energy use. Further it is highlighted that there are significant challenges and barriers, as well as a pressing need for air quality monitoring, coordinated policies and effective air quality management to ensure sustainable development, mitigate the adverse health impacts of pollution and improve the quality of life across the continent.

## Graphical abstract

Africa's world fastest growing population and unregulated rapid urbanization and industrialization are posing serious air quality challenges. This perspective discusses the barriers to effective air quality monitoring and regulation, highlighting the need for well‐designed frameworks to support sustainable development, improve public health, and reduce health inequalities across African megacities.

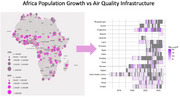

## 1 Background

Air pollution is the leading environmental risk to public health, causing more than 8 million premature deaths annually.^[^ ^1^ ^]^ Fine particulate matter (PM_2.5_), the major contributor to this burden, is causally linked to chronic diseases, asthma,^[^ ^2^ ^]^ tuberculosis, and child pneumonia.^[^ ^3^ ^]^ PM_2.5_ originates from vehicles, household fuel use, industry, dust, and microorganisms and can penetrate deep into the lungs and bloodstream, triggering oxidative stress, inflammation, and diseases including cancer.^[^ ^4^ ^]^ Africa faces disproportionate impacts; in 2019, air pollution caused 1.1 million deaths, surpassing the combined toll of malaria, Human Immunodeficiency Virus (HIV) and tuberculosis.^[^ ^5^ ^]^ Exposure to PM_2.5_ has been associated with 449 000 annual preventable childhood deaths in sub‐Saharan Africa, with projections suggesting that it could account for more than 30% of infant deaths in the coming years.^[^ ^6^ ^]^ The main reason for this excess burden of air pollution‐related mortality is the region's reliance on polluting solid fuels and kerosene for household energy, relied on by more than two‐thirds of the population. Household air pollution (HAP) from the combustion of these fuels accounts for almost two‐thirds of the entire burden of air pollution whereas ambient air pollution represents one‐third. However, these percentages are likely to change soon due to the projected rapid urbanization and industrialization in the region.

Population growth from urbanization is increasing the occurrence of serious air pollution health outcomes, with most major cities worldwide experiencing air pollution levels that exceed World Health Organization (WHO) air quality standards.^[^ ^7^ ^]^ Africa is a region of major concern due to its status as the world's second‐largest continent, home to the second‐largest global population with rapidly growing trends. The continent's urban areas are projected to see a massive increase in population in the coming decades. By 2100, 13 of the 20 largest megacities, cities with populations exceeding 10 million, will be in Africa. Megacities such as Lagos, Cairo, and Kinshasa are experiencing accelerated and unplanned growth, facing difficulties in policy regulation and monitoring infrastructure, which have resulted in significant increases in air pollution.^[^ ^8^ ^]^

This issue is further exacerbated by conflicting institutional interests. Governments, Non‐Government Oganizations (NGOs), and stakeholders often struggle to align their priorities regarding air quality management, leading to fragmented efforts.^[^ ^9^ ^]^ A lack of reliable monitoring data further hampers the ability to implement effective air quality policies or to estimate the burden of diseases caused or exacerbated by air pollution. Researchers and policymakers have traditionally relied on limited recordings of air quality that are publicly available from reference monitors such as those located at all urban US Embassy locations, which provide real‐time continuous data on PM_2.5_. Database which has become increasingly inconsistent due to data gaps (see Section 3). Other data sources include proxies such as airport visibility data to assess historical air pollution trends. For example, visibility data analyses, spanning the last five decades across the capitals of Kenya, Uganda, and Ethiopia, have shown 50–250% increase in ambient air pollution.^[^ ^10^ ^]^ However, such proxies are likely to suffer from inaccuracy leading to misclassification of exposure levels and underestimate or overestimate the true magnitude of air pollution trends and associated health impacts.

The urgency for enhanced air quality management in these growing urban areas cannot be overstated.^[^ ^11^ ^]^ Over 80% of air quality control strategies in Africa focus on HAP, while only 17% focus on ambient sources of air pollution, yet these industrial, traffic, and trash‐burning sources are increasing as urbanization increases.^[^ ^12^ ^]^ Whilst air quality control strategies in many high‐income cities take a multifaceted approach that accounts for sources, emissions, chemical transformations, and meteorological effects, these elements remain underutilized and poorly understood in the fast‐growing, unevenly expanding cities of emerging economies. This gap in failing to put knowledge into action underscores the importance of improving air quality frameworks to mitigate the adverse health effects of pollution in these regions and raise questions about the burden of diseases from exposure to air pollution in the African region.

## 2 Key Drivers of Air Pollution in African Megacities

### 2.1 Urbanization

Urbanization significantly contributes to air pollution in African megacities by increasing population density and associated anthropogenic emissions. Urbanization and economic prosperity are closely linked as expanding African economies often trigger a surge in urban populations. The better employment prospects, education, and healthcare in urban areas draw people from rural regions into cities. As of 2018, Cairo, Kinshasa, Lagos and greater Johannesburg were Africa's only megacities, but Dar es Salaam and Luanda are projected to surpass 10 million people by 2030. The number of cities with 5–10 million people is also expected to rise from five to thirteen,^[^ ^13^ ^]^ whereas over half of the projected global population increase between 2020 and 2050 and 90% from 2050 to 2100 is expected to occur in sub‐Saharan Africa, where the population is projected to triple by 2100.^[^ ^14^, ^15^ ^]^ As urban areas expand, the demand for infrastructure and services rises, leading to higher emissions from transportation, industry, and energy production, which in turn deteriorates air quality.^[^ ^16^ ^]^ Rapid urban growth presents challenges in managing air pollution, exacerbating pollution levels and health risks associated with poor air quality.^[^ ^17^ ^]^ The unique urbanization challenges such as cities’ topography, complex terrain and socio‐economic factors, complicate efforts to manage air pollution effectively,^[^ ^18^, ^19^ ^]^ while case studies or data regarding the extent of air pollution in African megacities are missing from the literature. As is often the case, rapid urbanization is met with the challenge of poor policy prioritization, where air pollution takes a back seat to issues like housing and poverty alleviation.

### 2.2 Industrialization

Industrialization is a major driver of air pollution in African megacities. Industrial activity projections until 2035, show that Cairo, Johannesburg, Lagos, Cape Town, and Alexandria have a steady compound annual growth rate of 4–5%. Cities like Kinshasa, Luanda, Nairobi, Douala, Abuja, and Kumasi will grow faster at 6–8%, while cities such as Dar es Salaam, Addis Ababa, Abidjan, and Kampala, will see growth rates near or above 10%.^[^ ^20^ ^]^ The growth of industry leads to increased emissions of pollutants such as sulfur dioxide (SO_2_) and nitrogen oxides (NO_x_), which contribute to poor air quality and can lead to severe environmental and public health challenges. In countries such as Nigeria, South Africa, Kenya, and Botswana, industrial growth, particularly in oil production, iron and steel industries, and urban‐based manufacturing, has led to sharp increases in emissions of particulate matter and secondary (formed in the atmosphere) aerosols.^[^ ^21^ ^]^ Nigeria's oil‐driven industrialization has heightened particulate matter pollution, while South Africa's iron and steel industries are major air pollution contributors.^[^ ^22^, ^23^ ^]^ Unregulated industries, such as brick kilns and small manufacturing plants, further degrade air quality, creating city‐wide hotspots, as seen in Lagos,^[^ ^8^ ^]^ while industrial pollution is a particular concern in cities like Accra, where industrial clusters near residential areas exacerbate PM_2.5_ levels.^[^ ^5^ ^]^ Industrial activities in African cities contribute 11–37% of direct PM_2.5_ levels which often exceed 150 µg m^−3^,^[^ ^24^ ^]^ depending on the size of the city and the type of industry.^[^ ^21^, ^25^, ^26^ ^]^ However, their overall contribution to poor air quality is likely greater due to associated emissions from waste and biomass burning. Addressing these air pollution issues requires robust policies and interventions, such as stricter emission standards and the mandatory installation of air pollution control technologies, including scrubbers in industrial factories located in urban settlements.^[^ ^11^ ^]^ However, despite the severity of the problem, there is limited information on the specific policies currently being implemented to tackle industrial air pollution across the continent.

### 2.3 Transportation

Transportation is a significant driver of air pollution in African megacities, largely due to emissions from vehicular traffic. These emissions include pollutants such as Coarse particulate matteer (PM_10_) and PM_2.5_, NO_2_, Carbon Monoxide (CO), Carbon Dioxide (CO_2_) and are exacerbated by the large concentration of vehicles in cities, traffic congestion, obsolescence of the vehicle fleet, and poor quality of fuels. Vehicle emissions, including exhaust and non‐exhaust sources like road dust resuspension, brake wear, and tire abrasion, are worsening urban air pollution, particularly in sub‐Saharan Africa, contributing to serious health issues and environmental degradation.^[^ ^27^ ^]^ Road dust resuspension, caused by the movement of vehicles, is a significant non‐exhaust source of PM, contributing substantially to PM_10_ and PM_2.5_ in urban areas, especially in unpaved or poorly maintained roads.^[^ ^28^ ^]^ These particles not only represent a health risk in themselves but also carry health‐damaging heavy metals, exacerbating health risks.^[^ ^29^ ^]^ In addition to PM, vehicle emissions include volatile organic compounds (VOCs). VOCs are key precursors to ozone (O_3_) formation, which is a short‐lived climate forcer and further degrades air quality, while VOCs such as benzene are known carcinogens. Quite concerningly, benzene levels around Nairobi and Lagos roadsides, for example, have been documented to be 4.3–4.8 times greater than the EU limit for health of 5 µg m^−3^.^[^ ^30^ ^]^ In many sub‐Saharan African cities, motorcycles are commonly used as motorcycle taxis and emit significant amounts of VOCs such as toluene and xylene, which contribute to ground‐level ozone formation. These vehicles have become an essential mode of transport, representing two‐thirds of all traffic in African cities.^[^ ^31^ ^]^ Traffic congestion also worsens air quality, where older vehicles using diesel and gasoline with poorly regulated exhaust emissions significantly contribute to air pollution.^[^ ^32^ ^]^ Traffic congestion in most African cities is greater than 26 min, with Lagos being the most congested city globally with 70 min.^[^ ^33^ ^]^ The United Nations Environmental Program (UNEP) estimates that Africa imports around 40% of the world's second‐hand light‐duty vehicles, contributing significantly to transportation‐related emissions due to outdated abatement technologies. Most African countries have no age restrictions on imported vehicles, many of which lack emissions controls or meet only outdated Euro 3 standards, far below the current Euro 6 standards. Poor fuel standards and ageing vehicle fleets are also key contributors to deteriorating transport emissions, with more than 50% of African countries having fuel quality worse than European standards pre‐dating 1992.^[^ ^34^ ^]^ Fuel sulfur levels in Africa often exceed 50 ppm, with some countries using fuels containing 2000–5000 ppm, compared to the 15 ppm limit required under Euro 6 regulations.^[^ ^35^ ^]^

### 2.4 Household Energy for Cooking, Heating, and Lighting

By far most households (some 70%) in sub‐Saharan Africa rely on polluting solid fuels (e.g., wood, charcoal, and biomass) and kerosene for household energy. Whilst this is a particular concern for peri‐urban and rural settings, in African cities charcoal and kerosene are still important sources of energy for cooking and lighting and are relied on by up to a third of households. The combustion of these fuels is a major contributor to both HAP and ambient air pollution in African megacities.^[^ ^36^ ^]^ Research has shown levels of PM_2.5_, and CO, significantly higher than WHO interim target levels for health with levels greater than 380 µg m^−3^ both in the home and for individuals;^[^ ^37^, ^38^ ^]^ often reaching 1000 µg m^−3^ in homes Nigeria.^[^ ^39^ ^]^ Women and children are particularly vulnerable to this exposure due to gender‐based meal preparation roles. HAP has a significant public health burden, leading to an estimated 742 000 premature deaths each year in sub‐Saharan Africa.^[^ ^3^ ^]^ HAP is exacerbated by the increasing use of diesel generators, which are required due to the unreliability of on‐grid electricity in many African cities. Despite the increasing availability of renewable energy sources and technologies that can be used for household energy in sub‐Saharan Africa, such as electricity produced from wind and solar energy, more than two‐thirds of sub‐Saharan Africa will still be without access to clean cooking fuels by 2030. Therefore, HAP is likely to remain a significant issue in sub‐Saharan African megacities for the foreseeable future.

### 2.5 Open Biomass and Waste Burning

In addition to HAP, open burning of biomass and non‐biomass waste is a significant source of ambient air pollution in African megacities. This practice, commonly used for managing household and municipal solid waste, releases harmful pollutants including PM_2.5_, CO, and NO_x_ affecting the health of residents near the burning sites. Biomass burning in West Africa during monsoon increases NO_x_, CO, and O_3_ by 7.98%, 11.5%, and 16%.^[^ ^40^ ^]^ Agricultural biomass burning, particularly during the dry seasons leads to sharp increases in PM_2.5_ and CO levels, with cities like Lagos and Abidjan experiencing heavy pollution from transboundary biomass burning in central and southern Africa.^[^ ^41^ ^]^ Open biomass burning is driven by agricultural practices such as burning fields and bushes in the postharvest season for fertilization, land management, and pest control. Biomass burning during the dry season has shown substantial increases in PM_2.5_ concentrations, significantly impacting air quality.^[^ ^42^ ^]^ Biomass burning contributes 15–39% to PM_2.5_ concentrations with PM_2.5_ and PM_10_ to be 39% greater during dry season.^[^ ^21^, ^43^, ^44^, ^45^ ^]^ For example, in Akure, Nigeria, PM_2.5_ concentrations during the dry season, heavily influenced by open burning, often exceeded 350 µg m^−^^3^, which is much higher than typical urban averages.^[^ ^46^ ^]^ Uncontrolled non‐biomass waste burning, further degrades air quality, emitting hazardous chemicals such as dioxins and furans from the combustion of organic and inorganic materials like plastics and tires. This practice contributes to both local and global air pollution.^[^ ^47^ ^]^ Additionally, waste management challenges, exacerbated by fragmented city policies and inadequate infrastructure, compound the problem. The combination of agricultural biomass burning, poor waste management, and traffic emissions leads to complex and compounded air quality issues in many urban and peri‐urban centres, while the associated economic costs of air pollution‐related productivity losses further underscore the urgent need for comprehensive air quality management policies.

## 3 Air Quality Regulation and Monitoring Infrastructure

**Figure 1:**
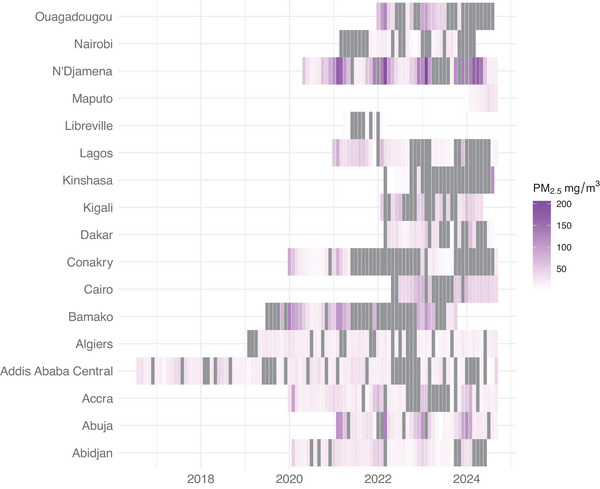
Monthly PM_2.5_ levels in selected African cities from 2017 until 2024. Grey colours indicate missing data. Data were acquired from US embassies (https://www.airnow.gov/international/us‐embassies‐and‐consulates/).

**Figure 2:**
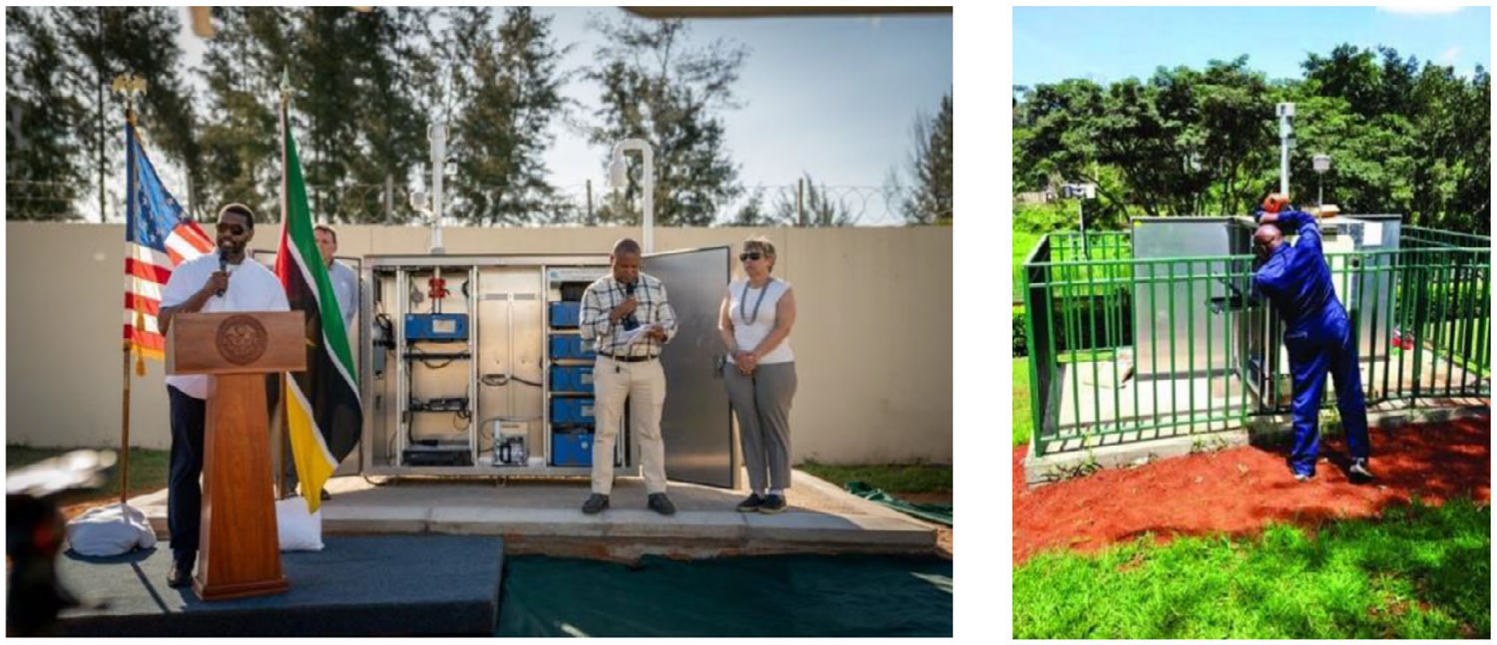
Air quality stations from US embassies in Africa (left in Maputo, Mozambique, right in Nairobi, Kenya).

Air quality monitoring is crucial for understanding and tracking air pollution levels to inform the implementation of mitigation policies and assess their impact. However, many African cities face serious obstacles in establishing efficient air quality monitoring systems. Most cities do not have the infrastructure, equipment and capacity needed to accurately measure air quality. Monitoring typically relies on single‐site measurements from facilities such as the US Embassy network, limited to one specific location in the capital city, which often experiences prolonged gaps in operation that result in significant data gaps (**Figure** **1**). Furthermore, the US Embassy reference monitors are located within the embassy premises, which are often surrounded by green space and trees that act as filters to reduce air pollution levels; hence, significantly underestimating actual pollution levels (**Figure** **2**).

Gaps in data collection significantly hinder efforts to develop and implement effective air pollution control strategies. The lack of monitoring systems stems from limited financial resources, a shortage of trained personnel, and an overall lack of technical expertise. In most African cities, air pollution monitoring tends to be limited and inconsistent, inhibiting the ability to identify peaks and longer‐term trends that result in delays or ineffective policy interventions.^[^ ^12^ ^]^

**Figure 3:**
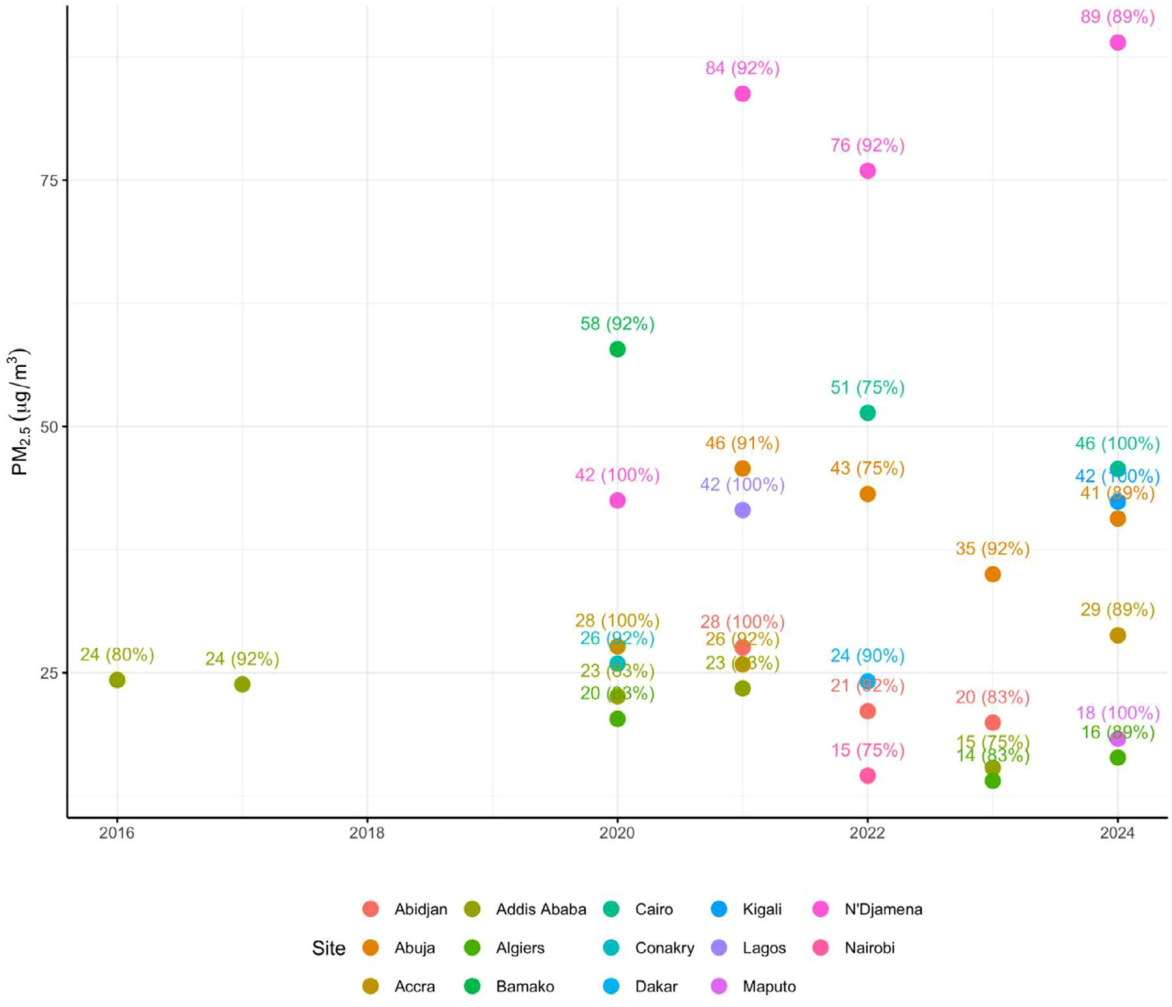
Annual PM_2.5_ levels across the US embassies in Africa. Values within brackets show data completion of that year. Valid were considered years that had at least 75% of data completion as per.^[^ ^50^, ^51^ ^]^

Initiatives such as the AirQo network^[^ ^48^ ^]^ have been launched to address this data shortage and are a step in the right direction. These initiatives aim to provide low‐cost air quality monitoring in multiple sites across an increasing number of African cities. The devices provide real‐time pollution data at a fraction of the cost of conventional reference monitoring stations. However, the cost savings from such monitors are often at the expense of data quality and reliability. For example, data drift following an initial calibration against reference standards requires routine calibrations to ensure accuracy, which can be prohibitively challenging to accomplish on a routine basis in these widespread networks of monitors. Satellite‐based PM_2.5_ estimates offer an alternative to ground monitoring but rely on algorithms converting aerosol optical depth (AOD) to PM_2.5_, which can be limited in Africa due to complex atmospheric conditions and varied emission sources.^[^ ^41^, ^49^ ^]^ The limited infrastructure and resources in African megacities severely restrict air quality monitoring efforts which consequently leads to limited air quality regulation. Whilst a priority for most African governments, enforcement of air quality regulations and standards is inhibited due to the limited financial and technical resources available. Although cities may adopt international air quality standards, they often lack the personnel, equipment, and funding required to monitor and enforce compliance. In 2017, the Lagos state government in Nigeria passed the Lagos State Environmental Management and Protection Law, whereas, in South Africa, the National Environmental Management: Air Quality Act stands out as one of the most comprehensive national frameworks for air quality management with clear standards for industrial emissions and regular monitoring. However, in many other African countries, air quality regulations are often outdated or not sufficiently enforced and often take a backseat in favor of development priorities and economic interests.^[^ ^27^ ^]^ WHO global guidelines set an annual limit for PM_2.5_ at 5 µg m^−3^ with an interim target 1 (WHO IT1) at 35 µg m^−3^. Out of the seventeen US embassies located in Africa, only fourteen have data and half of them do not meet the WHO IT1 (**Figure** **3**), which highlights the difficulty to assess the status of air quality in African megacities or emerging megacities and set national standards. NGOs and international organizations play a vital role in addressing air pollution across Africa. WHO and UNEP have been instrumental in providing financial and technical assistance to African nations, however, they often encounter barriers in policy integration because most nations have overlapping mandates and lack institutional coordination within and between government institutions at national and city levels.

## 4 Achieving Sustainable Development Goals Through Air Quality Improvements

Air pollution in African megacities presents a growing public health emergency and despite the rising PM_2.5_ levels, only 17 of the 54 African countries have established ambient air quality standards.^[^ ^52^, ^53^ ^]^ Inadequate air quality monitoring prevents accurate burden of disease estimates due to air pollution and further prevents progress toward several Sustainable Development Goals (SDGs), including SDG 3 (Good Health and Well‐being), SDG 7 (Access to Affordable and Clean Energy), SDG 11 (Sustainable Cities and Communities), and SDG 13 (Climate Action). As a result, cities like Accra, Cairo, Johannesburg, Lagos, and Nairobi, are projected to reach losses of nearly $140 billion by 2040.^[^ ^54^ ^]^

Informed decision‐making about mitigation strategies requires accurate data on air quality, but many cities lack the necessary equipment for real‐time monitoring, which hinders attempts to reduce negative effects on the environment and human health. Systematic data collection and research will help to better understand the sources and impacts of air pollution in the region. This includes long‐term air quality monitoring, health impact assessments, and multidisciplinary research involving local stakeholders. To achieve SDG 11, which calls for sustainable urbanization, it is imperative to invest in data collection tools. Expanding access to sustainable energy sources (in keeping with SDG 7) and encouraging the adoption of electric vehicles could greatly reduce air pollution. To keep in line with SDG 13, stricter regulations around industrial emissions, transportation, and waste management are required. Whereas to create air quality management strategies that reflect the unique challenges and context of African cities, such as the initiative between the World Bank and Lagos state,^[^ ^55^ ^]^ a collaborative, multi‐stakeholder bottom‐up approach is needed involving policymakers, civil society, local communities, and academics. By improving air quality, African megacities can reduce healthcare costs, increase life expectancy, and enhance economic productivity, all of which contribute to the SDG 3.

Air pollution is a global problem that requires local interventions and must be addressed with equitable resource management and distribution. Lessons from Asian megacities demonstrate how targeted policies and technological advancements can significantly improve air quality. For example, the Beijing Clean Air Action Plan (2013–2017) implemented measures to regulate urban development intensity, population size, vehicle ownership, and phase out coal consumption. These efforts led to reductions of 34% in PM_2.5_, 24% in PM_10_, 17% in NO_2_, 68% in SO_2_, and 33% in CO.^[^ ^56^ ^]^ Africa can adopt similar strategies to tackle air pollution while advancing progress toward the SDGs. Failing to act will prolong poor air quality conditions in the continent while worsening global environmental health inequalities.

## Conflict of Interest

The authors declare no conflict of interest.
